# Insights Into Walnut Lipid Metabolism From Metabolome and Transcriptome Analysis

**DOI:** 10.3389/fgene.2021.715731

**Published:** 2021-09-03

**Authors:** Suxian Yan, Xingsu Wang, Chenkang Yang, Junyou Wang, Ying Wang, Bangbang Wu, Ling Qiao, Jiajia Zhao, Pourkheirandish Mohammad, Xingwei Zheng, Jianguo Xu, Huming Zhi, Jun Zheng

**Affiliations:** ^1^State Key Laboratory of Sustainable Dryland Agriculture, Institute of Wheat Research, Shanxi Agricultural University, Linfen, China; ^2^College of Food Science, Shanxi Normal University, Linfen, China; ^3^Plant Molecular Biology and Biotechnology Laboratory, Faculty of Veterinary and Agricultural Sciences, University of Melbourne, Parkville, VIC, Australia

**Keywords:** walnut, lipidomic, UHPLC-Orbitrap HRMS, metabolism, transcriptome

## Abstract

Walnut oil is an excellent source of essential fatty acids. Systematic evaluation of walnut lipids has significance for the development of the nutritional and functional value of walnut. Ultra-performance liquid chromatography/Orbitrap high-resolution mass spectrometry (UHPLC-Orbitrap HRMS) was used to characterize the lipids of walnut. A total of 525 lipids were detected and triacylglycerols (TG) (18:2/18:2/18:3) and diacylglycerols (DG) (18:2/18:2) were the main glycerolipids present. Essential fatty acids, such as linoleic acid and linolenic acid, were the main DG and TG fatty acid chains. Many types of phospholipids were observed with phosphatidic acid being present in the highest concentration (5.58%). Using a combination of metabolome and transcriptome analysis, the present study mapped the main lipid metabolism pathway in walnut. These results may provide a theoretical basis for further study and specific gene targets to enable the development of walnut with increased oil content and modified fatty acid composition.

## Introduction

As one of the four major nut crop species in the world, walnut (*Juglans regia* L.) is widely distributed in Asia, Europe, North America, and Africa. In 2019, the world output of walnut was about 3.66 million tons (Zhou et al., 2018). The cultivated area and yield of walnut rank first among all types of dried fruits, and the crop has high economic value (Martínez et al., 2010). The oil content of walnut kernels is 52–70% and walnut oil is an excellent source of essential fatty acids with high nutritional value. Walnut kernels can be eaten fresh or dried. Dried walnuts are currently the most important walnut product. Less than 10% of dried walnuts are highly processed into walnut food. Walnut is also a good source of vegetable oil, which can be used for cooking and as an ingredient in paint and cosmetics (Zambón et al., 2000). The major constituents of walnut oil are triacylglycerols (TG) and diacylglycerols (DG). TGs are the major storage lipids and are an important energy reserve for the seed for germination and development (Shiu-Cheung and Randall, 2006). TG composition indicates the quality and purity of vegetable oils and is increasingly being used by the food industry to confirm oil authenticity. TG and DG constitute a good source of essential fatty acids of which linoleic acid and linolenic acid are the most common (Bouabdallah et al., 2014). In walnut oil, the ratio of n-3 and n-6 unsaturated fatty acids is 4∼6:1, which is in line with healthy dietary standards for humans (Croitoru et al., 2019). Therefore, it is vital to analyze and compare the lipid composition of walnut comprehensively from the perspective of the lipidome.

Lipids are currently classified into eight accepted categories by “The International Lipid Classification and Nomenclature Committee” as follows: fatty acyls (FA), glycerolipids (GL), glycerophospholipids (GP), sphingolipids (SP), saccharolipids (SL), sterol lipids, prenol lipids, and polyketides (Fahy et al., 2009). For lipidomic separation and investigation, thin-layer chromatography (TLC) was first used, and it has been gradually replaced by gas chromatography (GC) and liquid chromatography (LC) for lower resolution and sensitivity. The combination of GC/LC and mass spectrometry can efficiently separate and accurately detect lipid molecules. However, GC can only analyze small lipid molecules (e.g., fatty acids and tocopherols) that are thermally stable and sufficiently volatile, and long-chain unsaturated fatty acids are easily destroyed (Hamide et al., 2015). High-performance liquid chromatography, including high-performance liquid chromatography (HPLC), ultra-high-performance liquid chromatography (UHPLC), and two-dimensional HPLC (2D HPLC), can quickly achieve high efficiency separation (Li et al., 2011). Modern mass spectrometry (MS) mass analyzers offer very high mass resolution and mass accuracy, such as Fourier transform ion cyclotron resonance (FT-ICR) and Orbitrap and time of flight (TOF) (Lee and Yokomizo, 2018). At present, ultra-performance liquid chromatography-high-resolution mass spectrometry (UPLC-MS) is the most used analytical platform for the analysis of plant lipid metabolism. For instance, LC-ESI-MS was used to extend our understanding of the dynamic changes in lipid molecules in high oleic acid peanut at different development stages (Liu et al., 2019). Three hundred phospholipid molecules were detected by liquid chromatography-quadrupole time-of-flight mass spectrometry (LC-Q-TOF) in the seeds of *Eryngium maritimum* and *Cakile maritima* (Zitouni et al., 2016). A total of 165 phospholipids were separated by hydrophilic action chromatography-electrospray atomization-ion trap-time-of-flight mass spectrometry (HILIC-ESI-IT-TOF-MS) in six nut species (Song et al., 2018).

Transcriptomics can reflect the gene expression of cells, tissues, and organisms at a specific time and location (Qi et al., 2011). Many candidate genes related to lipid metabolism can be found with transcriptomics. For example, 4,817 differentially expressed genes were found from the dynamic changes of the transcriptome associated with oil accumulation at different developmental stages in walnut embryos. Among them, *ACCase*, *LACS*, and *FAD7* were identified as key genes for fatty acid synthesis (Zhao et al., 2018). Huang et al. (2021) found 108 genes related to lipid synthesis, including 60 genes for the fatty acid synthesis pathway, 33 for the triglyceride synthesis pathway, seven genes for the formation of oil bodies, and eight transcription factors. By analyzing the miRNA and mRNA transcriptome data of walnut kernels at different developmental stages, 104 miRNAs related to oil accumulation were found (Zhao et al., 2020). Lipid synthesis is the result of the interaction of a multilayer network. Single omics data cannot fully reflect the metabolic activity of cells. Multi-omics analysis is more robust. For example, Rothenberg et al. (2019) analyzed the molecular mechanisms driving anthocyanin accumulation in the development of mutant pink tea flowers (*Camellia sinensis* L.) by combining transcriptomics and metabolomics. Since multi-omics analysis can more clearly identify the genes regulating walnut oil metabolism, it was used in the present study.

UHPLC-Orbitrap HRMS was used to systematically compare the kernel lipid composition of different walnut varieties. The results provide a reference for studying walnut functional lipid components and improving the nutritional quality of walnuts.

## Materials and Methods

### Plant Materials

Xin 2, a precocious walnut variety, produces fruit early with high yield. However, Xin 2 has an astringent taste. The variety Qingxiang combines the advantages of precocious walnut and late walnut with long storage life and good quality (Wang et al., 2012). Walnut samples were collected in the XI county test station in China (110°57′E, 36°42′N, elevation 1,100 m, annual average precipitation 570 mm, annual average temperature 9.5°C). Normal plants were selected from orchards with stable yield. After harvest in mid-October, the green fruit husks were removed and washed. The fruits were then dried at 32°C for 10 h, at 37°C for 24 h, and at 35°C for 15 h before further analysis.

### Instruments and Reagents

The following were used in the experiments: UHPLC Nexera LC-30A ultra-high-performance liquid chromatograph (Shimadzu Co. Ltd., Tokyo, Japan), Q-Exactive mass spectrometer (Thermo Fisher Scientific, Waltham, MA, United States), low-temperature high-speed centrifuge (Eppendorf 5430R, Framingham, MA, United States), Acquity UPLC CSH C18 column (1.7 μm, 2.1 mm × 100 mm, Waters Corporation, Milford, MA, United States). Acetonitrile, isopropanol, methanol, methyl tert-butyl ether and 13 isotopic internal standards: Cer, LPC, PC, LPE, PE, PI, PS, PA, PG, SM, Chol Ester, DG, and TG (Thermo Fisher Scientific, Beijing, China).

### Sample Processing

Ten smooth, plump, uniform kernels each of Qingxiang and Xin 2 were selected. The embryos were frozen in liquid nitrogen and ground into a homogenized powder. Thirty milligrams of the powder was thoroughly mixed with 200 μl distilled water and 20 μl internal standard solution. Next, 800 μl of methyl tert-butyl ether and 240 μl of precooled methanol were added. A vortex mixer was used to agitate the mixture throughout the process. The samples were subjected to ultrasound mixing in cold water for 20 min and then allowed to stand at room temperature for 30 min. Samples were centrifuged at 14,000 × *g* at 10°C for 15 min and the upper organic phase was removed and blown dry with nitrogen. Before analysis, 200 μl of 90% isopropanol/acetonitrile solution was added to redissolve the samples, and 90 μl of the sample solution was centrifuged for 15 min at 14,000 × *g* and 10°C. Three microliters of the supernatant was used for analysis. All reagents used were chromatographically pure. Each sample was tested four times in succession.

### Chromatographic Conditions

The separation was performed on a UHPLC Nexera LC-30A. The chromatography column was at 45°C. The flow rate was 300 μl/min. Mobile phase A was acetonitrile–water solution (acetonitrile:water = 6:4, v/v) and phase B was acetonitrile–isopropanol solution (acetonitrile:isopropanol = 1:9, v/v). The gradient elution was programmed as follows: 0–2 min with 30% B, 2–25 min with 30–100% B, and 25–35 min with 30% B. The sample was placed in a 10°C automatic sampler for analysis.

### Mass Spectrometry Conditions

The samples were separated by UHPLC and analyzed by mass spectrometry with a Q Exactive mass spectrometer. Electrospray ionization (ESI) was performed in positive and negative ion modes. ESI source conditions were as follows: sheath gas flow rate 45 arb, auxiliary gas flow rate 15 arb, collision gas flow rate 1 arb, spray voltage 3.0 kV, capillary temperature 350°C, atomization temperature 300°C, S-Lens RF level 50%, and MS_1_ scanning range *m*/*z* 200–1,800. The mass charge ratio of lipid molecules and lipid fragments was obtained by collecting 10 fragment maps (MS_2_ scan, HCD) after each full scan. MS_1_ had a resolution of 70,000 at *m*/*z* 200 and MS_2_ had a resolution of 17,500 at *m*/*z* 200. The above experiments were completed by Applied Protein Technology Company.

### Data Analysis

The internal standard method was used for absolute quantification. The absolute content of the analyte was calculated by the response abundance ratio (peak area ratio) of the analyte and the internal standard when the concentration of the internal standard was known. Lipid data were obtained using Analyst^R^ TF 1.6 and Multi Quant^TM^ software (Taguchi and Ishikawa, 2010), and the peaks of lipid molecules and the internal standard lipid molecules were identified by LipidSearch. The main parameters were precursor tolerance 5 ppm, product tolerance 5 ppm, and product ion threshold 5%. The identification of lipid molecular species was mainly based on retention time, accurate *m*/*z*, and fragmentation ion patterns. Quantitative statistics and lipid composition analysis were performed with Microsoft Excel 2007 and Origin 8.5.

The original transcriptome sequencing data were obtained from Huang et al. (2021). The BioProject accession number of the data was PRJNA643637. Quality control of the downloaded transcriptome raw data was performed with FASTP V0.20.1 (Chen et al., 2018). After quality control, the clean data were aligned to a reference genome using HISAT2 (v2.0.5).^1^ FeatureCounts (Yang et al., 2014) was used for gene quantitative analysis. EggNOG V5.0 (Huerta-Cepas et al., 2017) was used for gene annotation.

## Results

### Data Quality Assessment

**FIGURE 1:**
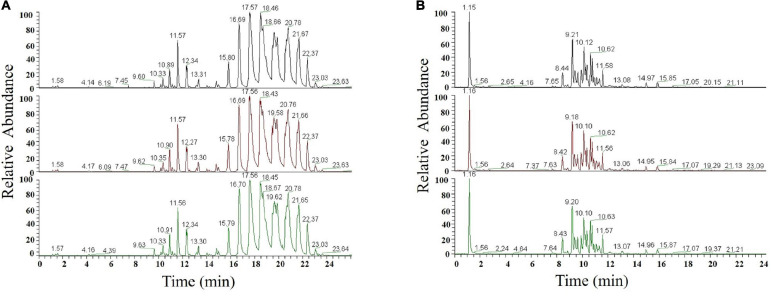
Chromatogram in ESI-positive **(A)** and ESI-negative ion modes **(B)**.

For each sample, good repeatability of the experiment was evidenced by both the response strength of the chromatographic peak and retention time being nearly identical between runs (Figure 1).

### Lipid Separation

**FIGURE 2:**
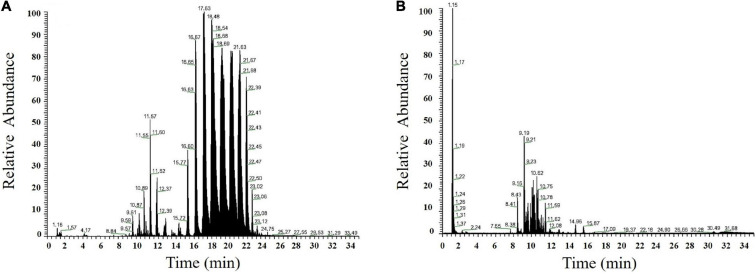
Chromatogram in ESI-positive **(A)** and ESI-negative ion modes **(B)** of XE.

The elution order of the same type of lipid molecules is determined by the number of carbon atoms and double bonds in the fatty acid chains. Retention time increased and elution slowed as the number of carbon atoms increased and vice versa. Nearly all sample peaks were detected within about 25 min. The peak shape, resolution, and response values were good. In the positive ion mode, glycerides (TG, DG) and some phospholipids (PC, PE, and LPC) had better mass spectrometry response intensity (Figure 2A). TG and DG generated primarily [M + NH_4_]^+^, PC, and PE, and LPC generated primarily [M + H]^+^. PI, PA, PS, PG, PIP, CL, LPE, LPG, LPI, some PE, PC, and saccharolipids had better responses under the negative ion mode (Figure 2B). PI, PA, PS, PG, PIP, PE, CL, LPE, LPG, and LPI produced primarily [M + H]^–^ and PC and glycolipid produced [M + HCOO]^–^.

For lipid identification, the databases LIPID MAPS^2^ and Lipid Bank^3^ were searched. In addition, walnut lipids can be distinguished by retention time in either positive or negative ion modes and by MS_1_ (primary mass spectrum) and MS_2_ (secondary mass spectrum) data. For example, the mass spectrum behavior of DG (18:2/18:2) can be explained as follows. The main mass spectral peak of DG (18:2/18:2) in positive ion mode was [M + NH_4_]^+^ (*m*/*z* 634.5405). The secondary mass spectrometry of DG generated fragmentation ions *m*/*z* 599.5035 and *m*/*z* 337.2734, corresponding to M-OH and NL [FA (18:2)-H + NH_4_]^+^, and generated characteristic fragmentation ion *m*/*z* 263.2367 after dissociation (Supplementary Figure 2A) corresponding to fatty acid C18:2. The molecule was identified as DG (18:2/18:2).

### Variance Analysis

Using partial least squares discrimination analysis (PLS-DA) (Supplementary Figure 3A), the model evaluation parameters (*R*^2^*Y*, *Q*^2^) obtained are listed in Supplementary Table 1. Generally, if *Q*^2^ is greater than 0.5, the model is the most stable and reliable; if 0.3 < *Q*^2^ ≤ 0.5, the model is stable; and if *Q*^2^ < 0.3, the reliability of the model is low.

**FIGURE 3:**
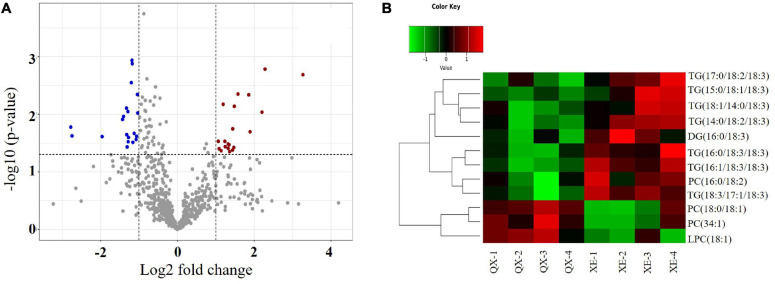
Analysis of differential lipid molecules in walnut seed kernels. **(A)** Volcano plot. The dots represent lipid molecules, among which the blue and red dots are differential lipid molecules that satisfies FC < 0.5, FC > 2, and *p* < 0.05. **(B)** Hierarchical and clustering analysis.

Difference analysis of all detected lipid molecules was performed and the results are expressed in a volcano graph (Figure 3A).

Of 525 lipid molecules in the two walnut samples, 12 molecules with significant differences (OPLS-DA variable importance for the projection > 1 and *p* < 0.05) were identified using the OPLS-DA model (Supplementary Figure 2B). Among these, there were seven TG molecules—TG (14:0/18:2/18:3), TG (18:1/14:0/18:3), TG (15:0/18:1/18:3), TG (16:1/18:3/18:3), TG (16:0/18:3/18:3), TG (18:3/17:1/18:3), and TG (17:0/18:2/18:3); one DG molecule—DG (16:0/18:3); three PC molecules—PC (34:1), PC (16:0/18: 2), and PC (18:0/18:1); and one LPC molecule—LPC (18:1) (Table 1).

**TABLE 1:** Lipid molecules showing significant differences between two walnut varieties (*p* < 0.05).

**Lipid group**	**Class**	**Fatty acid**	**Ion formula**	**Cal Mz**	**QX (μg/g) ± SD**	**XE (μg/g) ± SD**	***p*-value**
PC (34:1) + H	PC	(34:1)	C_42_ H_83_ O_8_ N_1_ P_1_	760.58	241.96 ± 75.77	119.26 ± 56.20	0.0337
TG (50:5) + NH_4_	TG	(14:0/18:2/18:3)	C_53_ H_96_ O_6_ N_1_	842.72	119.48 ± 16.05	181.00 ± 15.00	0.0052
TG (50:4) + NH_4_	TG	(18:1/14:0/18:3)	C_53_ H_98_ O_6_ N_1_	844.73	259.03 ± 42.34	381.81 ± 51.73	0.0382
TG (51:4) + NH_4_	TG	(15:0/18:1/18:3)	C_54_ H_100_ O_6_ N_1_	858.75	167.36 ± 9.22	235.70 ± 36.14	0.0344
TG (52:7) + NH_4_	TG	(16:1/18:3/18:3)	C_55_ H_96_ O_6_ N_1_	866.72	106.63 ± 22.35	200.55 ± 23.52	0.0024
TG (52:6) + NH_4_	TG	(16:0/18:3/18:3)	C_55_ H_98_ O_6_ N_1_	868.74	416.58 ± 125.62	751.36 ± 165.34	0.0204
TG (53:7) + NH_4_	TG	(17:1/18:3/18:3)	C_56_ H_98_ O_6_ N_1_	880.74	124.57 ± 23.16	274.53 ± 12.97	0.0050
TG (53:5) + NH_4_	TG	(17:0/18:2/18:3)	C_56_ H_102_ O_6_ N_1_	884.77	207.39 ± 58.96	382.70 ± 60.74	0.0252
DG (34:3) + NH_4_	DG	(16:0/18:3)	C_37_ H_70_ O_5_ N_1_	608.52	945.60 ± 310.82	1,443.94 ± 394.57	0.0352
LPC (18:1) + HCOO	LPC	(18:1)	C_27_ H_53_ O_9_ N_1_ P_1_	566.35	69.83 ± 14.88	16.00 ± 7.11	0.0178
PC (34:2) + HCOO	PC	(16:0/18:2)	C_43_ H_81_ O_10_ N_1_ P_1_	802.56	442.17 ± 83.06	535.81 ± 71.90	0.0494
PC (36:1) + HCOO	PC	(18:0/18:1)	C_45_ H_87_ O_10_ N_1_ P_1_	832.61	43.45 ± 7.17	9.50 ± 5.11	0.0327

*Cal Mz means mass-to-charge ratio. QX (μg/g) ± SD and XE (μg/g) ± SD are the means of four replicates ± standard deviation.*

Hierarchical clustering of the differential lipid molecules based on the expression levels showed that the contents of PC (34:1), PC (18:0/18:1), and LPC (18:1) were higher in Qingxiang, and the contents of TG (14:0/18:2/18:3), TG (18:1/14:0/18:3), TG (15:0/18:1/18:3), TG (16:1/18:3/18:3), TG (16:0/18:3/18:3), TG (18:3/17:1/18:3), TG (17:0/18:2/18:3), DG (16:0/18:3), and PC (16:0/18:2) were higher in Xin 2 (Figure 3B).

### Lipid Species in Walnut Kernels

**FIGURE 4:**
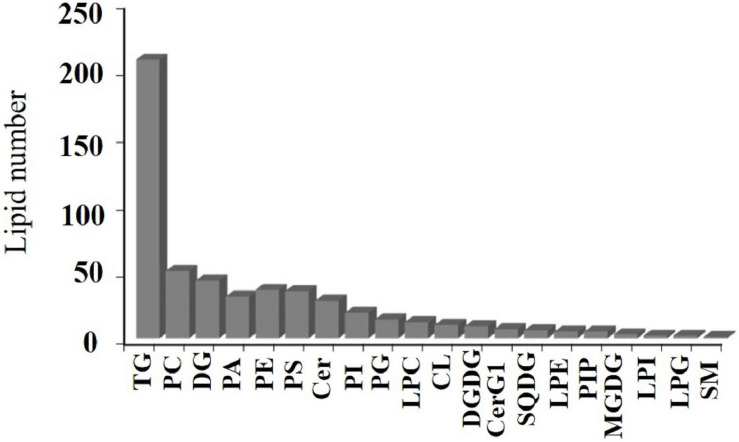
Walnut kernel lipid composition.

A total of 525 lipid molecules representing 20 lipid subclasses were identified. The number of lipid molecules in different subtypes varied greatly (Figure 4). A total of 250 types of GLs were detected, of which TG was most frequent, with 207 types of TGs, and DGs were the next most common with 43 types. There were 221 types of GPs consisting of 50 PCs, 31 PAs, 36 PEs, 35 PSs, 19 PIs, 14 PGs, 12 LPCs, 5 LPEs, 2 LPGs, 2 LPIs, 5 PIPs, and 10 CLs. There were 36 kinds of SPs, consisting of 28 Cers, 7 CerG1s, and 1 SM. There were 18 kinds of SLs, including 3 MGDGs, 3 DGDGs, and 6 SQDGs.

In addition, there was a rich array of fatty acids present, including 21 saturated fatty acids, namely, C4:0, C8:0, C10:0, C12:0, C13:0, C15:0, C14:0, C16: 0, C17:0, C18:0, C19:0, C20:0, C21:0, C22:0, C23:0, C24:0, C25:0, C26:0, C27:0, C29:0, and C30:0, and 27 unsaturated fatty acids, namely, C10:1, C10:2, C12:1, C14:1, C14:2, C14:3, C16:1, C17:1, C18:1, C18:2 C18:3, C18:4, C19:1, C20:1, C21:1, C20:2, C20:4, C20:5, C22:4, C22:5, C22:6, C24:1, C24: 2. C26:1, C28:1, C29:1, and C30:1. Some rare medium-chain fatty acids (C4:0, C8:0, C10:0, C10:1, C10:2) and ultra-long-chain fatty acids (C24:2, C25:0, C26:0, C27:0, C26:1, C28:1, C29:1, C30:1) were present.

### Lipid Content in Walnut Kernels

**FIGURE 5:**
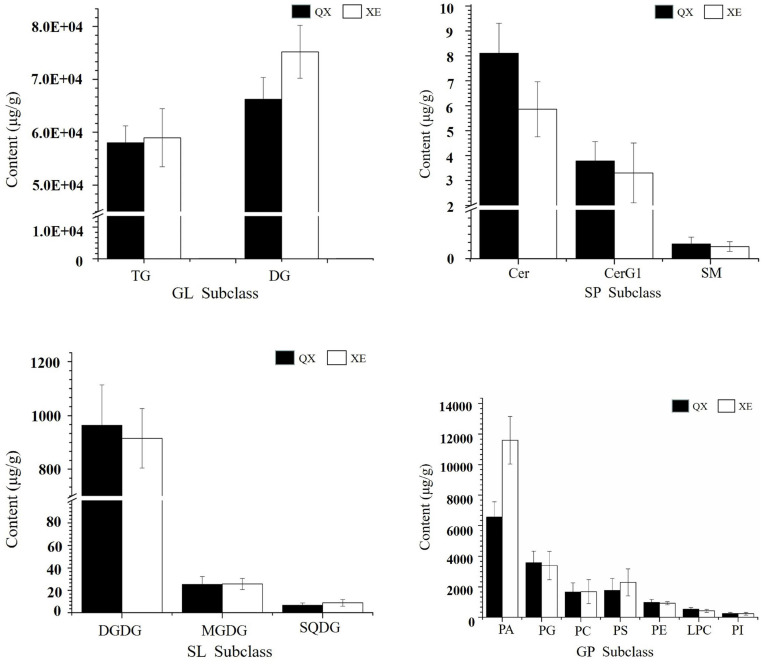
The content of lipid subtypes in two walnut varieties. The ordinate represents the sum of the lipid molecules with the same lipid subtypes.

Comparing the lipid content of the two walnut varieties, Qingxiang had 140,711 μg/g and Xin 2 had 155,801 μg/g. The content trends of the lipid components of Qingxiang and Xin 2 were nearly identical, with both having the highest content of glycerides (including DG and TG) accounting for 88.37 and 86.18% of the total lipid content, respectively. Phospholipids were the second most common type (including PA, PG, PS, PC, PE, LPC, PI, LPG, LPI, LPE, PIP, and CL) accounting for, respectively, 10.9 and 13.2% of the total lipids. Glycolipids (including DGDG, MGDG and SQDG) accounted for 0.7 and 0.61% and sphingolipids (including Cer and SM) accounted for 0.03 and 0.01%, respectively. In addition, comparing the lipid subtypes (Figure 5), the content of DG, TG, PA, and PS in Xin 2 was relatively high compared with that in Qingxiang.

**FIGURE 6:**
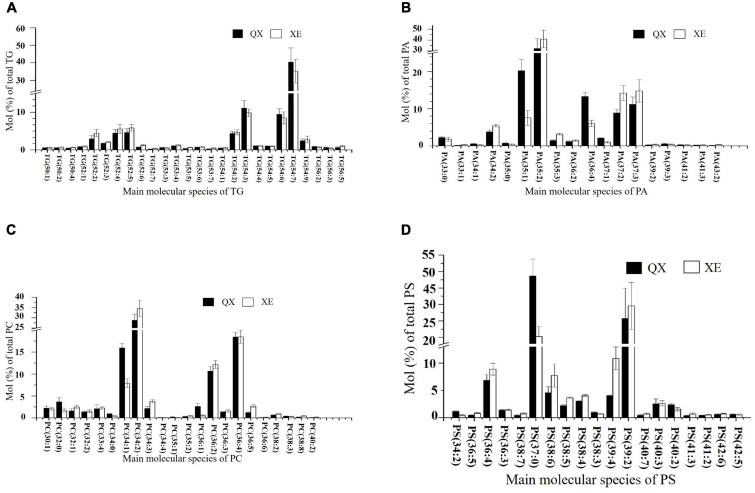
The content of the main lipid molecules in kernels of the two walnut varieties. Major molecular species composition of TG **(A)**, PA **(B)**, PC **(C)**, and PS **(D)** determined by UHPLC-Orbitrap HRMS in the walnut varieties Qingxiang and Xin 2.

In both walnut varieties, the main molecules among the TGs were TG 54:7 (18:2/18:2/18:3, LLLn), TG 54:6 (including TG 18:1/18:2/18:3, OLLn; and TG 18:2/18:2/18:2, LLL), TG 54:3 (18:1/18:1/18:1, OOO), TG 52:4 (16:0/18:1/18:3, POLn), and TG 52:5 (16:0/18:2/18:3, PLLn), with mainly four kinds of fatty acids: C16:0, C18:1, C18:2, and C18:3 (Figure 6A). TG (14:0/18:2/18:3), TG (18:1/14:0/18:3), TG (15:0/18:1/18:3), TG (16:1/18:3/18:3), TG (16:0/18:3/18:3), TG (18:3/17:1/18:3), and TG (17:0/18:2/18:3) (*p* < 0.05) were significantly higher in Xin 2. Linoleic acid (18:2) and linolenic acid (18:3) contents were higher in Qingxiang, whereas the palmitic acid content was greater in Xin 2 (P: palmitic acid; S: stearic acid; O: oleic acid; L: linoleic acid; Ln: linolenic acid).

Analysis of the degree of unsaturation of the TG molecules indicated that seven double bonds were the most common, thus showing a higher degree of unsaturation. Eight TG molecules had saturated carbon chains, but their content was lower (≤ 10 μg/g). The remainder of the TG molecules were unsaturated. This result shows that both Qingxiang and Xin 2 contain large amounts of unsaturated fatty acids.

In the TG molecules, 50–56 carbon atoms were present. The main fatty acids connected with TG were medium-chain C16, C17, and C18 and the content of long-chain fatty acids was low (≤ 10 μg/g). There were more C54 TG molecules in Qingxiang, whereas C52 TG molecules were slightly higher in Xin 2 (Supplementary Figure 3B).

Phospholipids were rich in walnut kernels, including PA, PG, PS, PC, PE, LPC, PI, LPE, CL, PIP, LPI, and LPG. The phospholipids in Qingxiang and Xin 2 accounted for approximately 10.9 and 13.2% of the total lipids, respectively. The phospholipid content in Xin 2 was relatively high. PA was the main phospholipid subtype, which accounted for 4.65% of the total lipids in Qingxiang and 7.45% in Xin 2. The main molecular species detected in Qingxiang were PA 35:2 (17:0/18:2) (32.23%), PA 35:1 (17:0/18:1) (20.24%), and PA 36:4 (18:2/18:2) (13.29%), while PA 35:2 (17:0/18:2) (40.91%), PA 37:2 (14.21%), and PA 37:2 (14.79%) were main PAs in Xin 2 (Figure 6B). In both walnut varieties, PA containing C16:0, C18:1, and C18:2 accounted for more than 50%, and a small content of long-chain fatty acids, such as 40:2, 40:3, 41:2, 41:3, 42:2, 43:2, and 44:3, was also detected.

There was a little difference in PC content between Qingxiang and Xin 2, accounting for 1.17 and 1.07% of the total lipids, respectively. The main molecules in PCs were PC 34:1 (16:0/18:1), PC 34:2 (16:0/18:2), PC 36:2 (18:0/18:2), and PC 36:4 (18:2/18:2). PC mainly contained fatty acids C16:0, C18:0, and C18:1 (Figure 6C). The content of PC 34:1 (16:0/18:1) (*p* = 0.0337) and PC (18:0/18:1) (*p* = 0.0494) was higher in Qingxiang and the content of PC 34:2 (16:0/18:2) (*p* = 0.0327) was higher in Xin 2.

PS contains amino groups, which have antioxidant effects. Of the total lipids present, PS accounted for about 1.25% in Qingxiang and for 1.47% in Xin 2. The main PS molecules were PS 37:0, PS 39:2, PS 36:4, and PS 39:4 (Figure 6D).

SL (DGDG, MGDG, SQDG) in Qingxiang and Xin 2 accounted for 0.7 and 0.61% of the total lipids, respectively. DGDG was the main component of SL, accounting for 76.32 and 65.23% in Qingxiang and Xin 2, respectively. The more abundant molecules were DGDG (18:2/18:2), DGDG (18:2/18:3), and SQDG (39:12). Saccharolipids are the main components of the membrane lipid in walnut, although the content is relatively small. Saccharolipids have a variety of pharmacological functions, such as antiviral, antioxidant, antitumor, and anti-atherosclerosis activities (Schinitz and Ruebsaamen, 2010).

The SP in Qingxiang and Xin 2 walnut kernels accounted for only 0.03 and 0.01% of total lipids, respectively. The contents of Cer (d32:0) and Cer (d34:0) were higher in SP (including Cer and SM). As a secondary signal molecule of cells, SP promotes cell proliferation, apoptosis, and growth arrest; inhibits the occurrence and metastasis of tumors; and increases the sensitivity of tumors to chemotherapeutic drugs (Goldkorn et al., 2013). A small amount of sphingomyelin SM (d22:1) was detected in the two walnut lipids.

### Lipid Metabolism of Walnut Analysis

**FIGURE 7:**
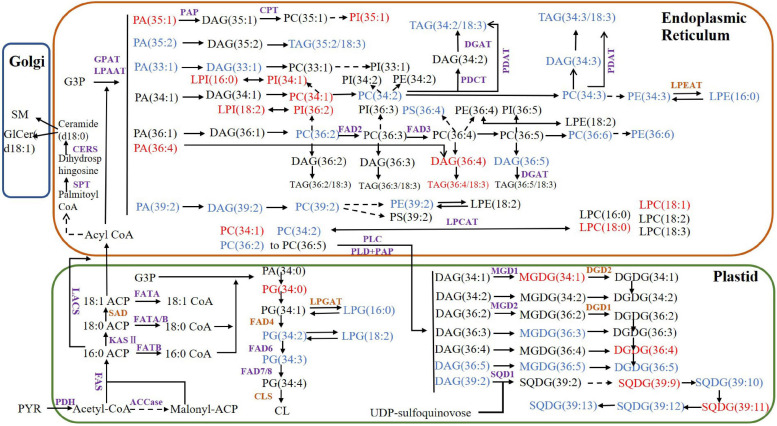
Proposed lipid metabolism pathway map for walnut. The purple enzymes represent genes that have been detected in the transcriptome and the brown genes represent the undetected genes. The lipid molecules in blue have higher content of Xin 2 and those in red have higher content of Qingxiang. PYR, pyruvate; ACP, acyl carrier protein; PDH, pyruvate dehydrogenase; ACCase, acetyl-CoA carboxylase; acyl CoA, acetyl-coenzyme A; FAS, fatty acid synthase; SAD, stearoyl-ACP desaturase; LACS, long-chain acyl-CoA synthetase; CPT, CDP-choline:diacylglycerol cholinephosphotransferase; KASII: 3-ketoacyl-ACP synthase; DGAT, diacylglycerol acyltransferase; FAD2, oleoyl desaturase; FAD3, linoleoyl desaturase; FAD4, FAD6, FAD7/8, fatty acid desaturase; GAP, glyceraldehyde-3-phosphate; GPAT, glycerol-3-phosphate acyltransferase; LPAAT, lysophosphatidic acid acyltransferase; PDAT, phospholipid:diacylglycerol acyltransferase; PDCT, phosphatidylcholine:diacylglycerol cholinephosphotransferase; PEP, phosphoenolpyruvate; MGD, monogalactosyldiacylglycerol synthase; DGD, digalactosyldiacylglycerol synthase; SQD, sulfoquinovosyldiacylglycerol synthase; CLS, cardiolipin synthase; SPT, serine palmitoyltransferase; CERS, ceramide synthase; SMS, sphingomyelin synthase; LPEAT, lysophosphatidylethanolamine acyltransferase; LPCAT, lysophosphatidylcholine acyltransferase; PLC, phospholipase C; PLD, phospholipase D; PAP, phosphatidic acid phosphatase.

Using the functional annotations of the expressed genes, the lipid metabolism-related genes in the transcriptome were identified. The proposed walnut lipid metabolism pathway map was generated corresponding to the main lipid molecules in the lipidome (Figure 7). A higher content of lipid molecules was detected in the lipid group, such as TG (36:4/18:3), TG (34:2/18:3), and TG (34:3/18:3). DGAT and PDAT related to TG synthesis were expressed in the transcriptome. In addition, there were many oleic acids (18:1) and the expression of PDH and ACCase related to oleic acid synthesis was also high. The content of lysophospholipids, saccharolipids, and sphingolipids was low, and the expression of the corresponding synthetase genes was either low (CERS and MGD1) or undetected (LPGAT, LPGAT, and DGD) (Supplementary Table 2). Genes with high expression levels in the transcriptome corresponded to high levels of lipid metabolism molecules. Some genes related to lipid metabolism molecules with lower content were not detected in the transcriptome. Perhaps, low abundance RNA was below the detection sensitivity of our methods, or perhaps, an undescribed gene was present which would require further study.

Comparing the lipid data of the walnut varieties, the content of glyceride TG (18:2/18:2/18:3) was the highest in two kinds of walnuts, while the contents of TG (15:0/18:1/18:3), TG (16:1/18:3/18:3), TG (16:0/18:3/18:3), TG (17:0/18:2/18:3), TG (16:0/18:3/18:3) were higher in Xin 2. The fatty acid composition differed significantly between the oils of Qingxiang and Xin 2. TG synthesis in walnut has two pathways. The Kennedy pathway relies on acyl-CoA. The three acyltransferases (GPAT, LPAT, and DGAT) transfer the fatty acids of acyl-CoA to glycerol. Another pathway uses direct transfer of FA from PC to DG producing TG and LPC by the PDAT (Bates et al., 2013). Both DGAT and PDAT cooperated to produce TG. DGAT and PDAT can be used as target genes to regulate the oil content of walnut through molecular technology.

DG (36:4) had relatively higher content in the two kinds of walnuts, whereas DG (34:2) and DG (34:3) contents were relatively higher in Xin 2. The phospholipids were mostly C36 molecules. PA and DG are important intermediate products in lipid metabolism. Their synthesis starts with G-3-P and fatty acids as initial substrates and includes the endoplasmic reticulum pathway (eukaryotic pathway) and the plastid pathway (prokaryotic pathway), which occur in different subcellular locations (Hong et al., 2017). The sn-2 position of glycerolipid molecules synthesized by the prokaryotic pathway generally prefers C16:0, while the sn-2 position of the lipids derived from the eukaryotic pathway is C18:1 (Schmid-Siegert et al., 2016). There were many C18 fatty acids in the lipid molecules of Qingxiang and Xin 2, thus showing that the eukaryotic pathway is the primary pathway of glycerolipid synthesis in walnut. The analysis also found that MGDG and DGDG in the walnut lipid were mainly 36:5, indicating that the intermediate products DG and PA produced by the ER pathway were likely the main substrate sources of MGDG and DGDG.

## Discussion

### Comparative Analysis of Lipid Composition

As an important oil tree species, walnut has high economic and nutritional value. Compared with other main nut crops, such as pistachios, cashews, peanuts, pecans, and almonds, walnuts have the most abundant phospholipids, accounting for 96 species (Song et al., 2018). Triglycerides were detected in the oils of walnut, sesame, water chestnut, hazelnut, and beechnut, with walnut oil mainly composed of highly unsaturated TG (54:6–8) (Bail et al., 2009). Research on the lipid composition of walnuts has been limited to the identification of the composition and content of single lipids such as fatty acids, phospholipids, and glycerides, and a systematic comparison of the total lipid composition of walnuts has not been done previously. The present study systematically analyzed and compared the lipid composition of the walnut varieties Qingxiang and Xin 2 and found a total of 525 lipid molecules in 20 subtypes. The lipid molecule contains 21 species of saturated fatty acids and 27 species of unsaturated fatty acids, including a low content of rare ultra-long-chain unsaturated fatty acids. The presence of these fatty acids indicates that special fatty acids dehydrogenase and elongase enzymes were likely responsible for the unsaturation and elongation of the glyceride chain (Ivanova et al., 2016).

There were more C54 TG molecules in Qingxiang than in Xin 2, and the C52 TG molecules in Xin 2 were slightly higher than in Qingxiang, both of which connected medium-chain fatty acids. Compared with long-chain fatty acid glycerides, medium-chain fatty acid triglycerides (MCT) in oils are easier to hydrolyze to produce unsaturated fatty acids. These fatty acids, which are absorbed easily by the body, can effectively reduce the levels of triglycerides and apolipoproteins and improve lipid metabolism (Fink et al., 2014). The unsaturation of TG molecules in Qingxiang was higher with greater linoleic acid and linolenic acid content. The quality of walnut oil mainly lies in the fact that it contains a large amount of unsaturated fatty acids, which can effectively reduce and prevent the occurrence of cholesterol, atherosclerosis, and heart disease (Ibáñez et al., 2017). Moreover, the oxidized linoleic acid will produce n-butyraldehyde and other volatile components that determine the flavor and taste of walnuts (Zhou et al., 2017). Qingxiang has good taste and high nutritional value, but its unsaturated fatty acids are oxidized easily, thus could reduce the shelf life of kernels and oil (Emilio and Mataix, 2006).

Twelve kinds of phospholipids were found in walnut oil encompassing 221 phospholipid molecules, which were the most abundant species. PA is a lipid signaling molecule that participates in various physiological processes, including signal transmission and response to environmental stress. Yu et al. (2010) found that under drought stress, PA 34:2, 34:3, 34:6, 36:3, and 36:6 increase significantly. The PA accounted for 7.45% in Xin 2, a relatively high content, which shows that Xin 2 is more resistant and adaptable. PG is rich in membrane lipids and is a biologically active lipid with antioxidant effects (Yang et al., 2007). PG (44:0) in Qingxiang and Xin 2 accounted for 98.37 and 97.44% of the total PG, respectively. These phospholipid molecules containing long-chain saturated fatty acids have antioxidant effects. The PS content in the Qingxiang and Xin 2 is low. However, PS contains amino groups, which can have synergistic antioxidant effects with vitamin E (Shen et al., 2013).

### Lipidome-Combined Transcriptome Analysis

By combining the analysis of expressed genes and lipid metabolism molecules, preliminary metabolic pathways of the main lipid molecules in walnuts were constructed. Qingxiang contained more linoleic acid (18:2), while Xin 2 contained more palmitic acid (16:0) and long-chain fatty acids such as behenic acid. FAD2 and FAD3 control, respectively, the conversion of oleic acid to linoleic acid and linoleic acid to linolenic acid (Liu et al., 2020). The expression levels of FAD2 and FAD3 in the transcriptome were higher than those of FAD6 and FAD7/8. Primarily, linoleic and linolenic acids were found in the present study and their generation may have been catalyzed by FAD2 and FAD3 in the ER. ACCase is the key rate-limiting enzyme for the assembly of fatty acids. Analysis of the transcriptome showed that ACC-1 and ACC-2 expressed higher levels, which is likely related to the high oil content in walnut kernels. The genes of FATA and FATB were also expressed, with that of FATA being the higher of the two. These relative expression levels are likely the reason why many unsaturated C18 fatty acids were present.

The molecular composition of TG differed greatly between the two walnut varieties. Both DGAT1 and DGAT2 in the transcriptome were expressed in varieties. A previous work has shown that they may play different roles during plant development and produce TG with different fatty acid components (Oakes et al., 2011). It is likely that these two enzymes are related to the varietal differences in TG species observed in the preset study. Furthermore, PDAT has different expression levels during walnut kernel development, which may be related to the accumulation of walnut oil (Bernard et al., 2018).

## Conclusion

In the present study, the UHPLC-Orbitrap HRMS system was used to compare the lipid content and composition in the kernels of the walnut varieties Qingxiang and Xin 2. Combined with transcriptome data, we constructed a preliminary molecular regulatory network of the main lipid metabolism in walnut. A total of 525 lipid molecules were identified in Qingxiang and Xin 2, consisting of 250 glycerides (including DG and TG), 221 phospholipids (including PA, PG, PS, PC, PE, LPC, PI, LPG, LPI, LPE, PIP, and CL), 18 types of glycolipids (including DGDG, MGDG, and SQDG), and 36 types of sphingolipids (including Cer and SM). The fatty acid chains in DG and TG are mainly composed of essential fats such as oleic acid, linoleic acid, and linolenic acid. The walnut lipid profile and the lipid metabolism pathway constructed here have important theoretical and practical value for further study of walnut lipid metabolism and functional development.

## Data Availability Statement

The datasets presented in this study can be found in online repositories. The names of the repository/repositories and accession number(s) can be found below: https://www.ebi.ac.uk/metabolights/index, MTBLS2888.

## Author Contributions

JZhe and JX designed the experiment. CY, JW, YW, HZ, BW, XW, LQ, and JZha performed the analysis and interpretation of the data. SY and XZ drafted the manuscript. JZhe and PM revised the manuscript. All authors contributed to the article and approved the submitted version.

## Conflict of Interest

The authors declare that the research was conducted in the absence of any commercial or financial relationships that could be construed as a potential conflict of interest.

## Publisher’s Note

All claims expressed in this article are solely those of the authors and do not necessarily represent those of their affiliated organizations, or those of the publisher, the editors and the reviewers. Any product that may be evaluated in this article, or claim that may be made by its manufacturer, is not guaranteed or endorsed by the publisher.

## Footnotes

1
http://daehwankimlab.github.io/hisat2/

2
https://www.lipidmaps.org/

3
http://www.lipidbank.jp/

## Supplementary Material

Supplementary Figure 1 Chromatogram in ESI positive **(A)** and negative ion modes **(B)** of QX.

Supplementary Figure 2 **(A)** Secondary mass spectrometry of DG (18:2/18:2) (m/z 634.5405) generated fragmentation ion; **(B)** bubble chart, bubble in figure present significant difference of lipid molecules, different colors present different lipid subclasses.

Supplementary Figure 3 **(A)** Score and loading plots generated from PLS-DA classifying the lipid of QX and XE. **(B)** The content of different numbers of carbon in TGs of two walnut varieties.

Supplementary Table 1 Model evaluation parameters: R^2^X means the explanatory rate for the model of variable X; R^2^Y means the explanatory rate for the model of variable Y; Q^2^ evaluates the predictive power of PLS-DA model.

Supplementary Table 2 The lipid molecules in lipid metabolism pathway of walnut kernel. QX (μg/g) and XE (μg/g) are the means four replicates ± standard deviation.

Supplementary Table 3 The enzymes involved in main lipid metabolism pathway of walnut kernel. FPKM means the genes of these enzymes expression level. ND means the genes were not detected.

## Abbreviations

DG: diacylglycerol
TG: triacylglycerol
PA: phosphatidic acid
PG: phosphatidylglycerol
PS: phosphatidylserine
PC: phosphatidylcholine
PE: phosphatidylethanolamine
PI: phosphatidylinositol
PIP: phosphatidylinositol diphosphate
LPC: lysophosphatidylcholine
LPE: lysophosphatidylethanolamine
LPG: lysophosphatidylglycerol
LPI: lysophosphatidylinositol
CL: cardiolipin
Cer: ceramide
SM: sphingomyelin
MGDG: monogalactosyldiacylglycerol
DGDG: digalactosyldiacylglycerol
SQDG: sulfoquinovosyldiacylglycerol.
