# Supplementary figures and images for: Insights Into Walnut Lipid Metabolism From Metabolome and Transcriptome Analysis
Source: Front Genet. 2021 Sep 3;12:715731. doi: 10.3389/fgene.2021.715731 (PMC8446449; doi:10.3389/fgene.2021.715731)

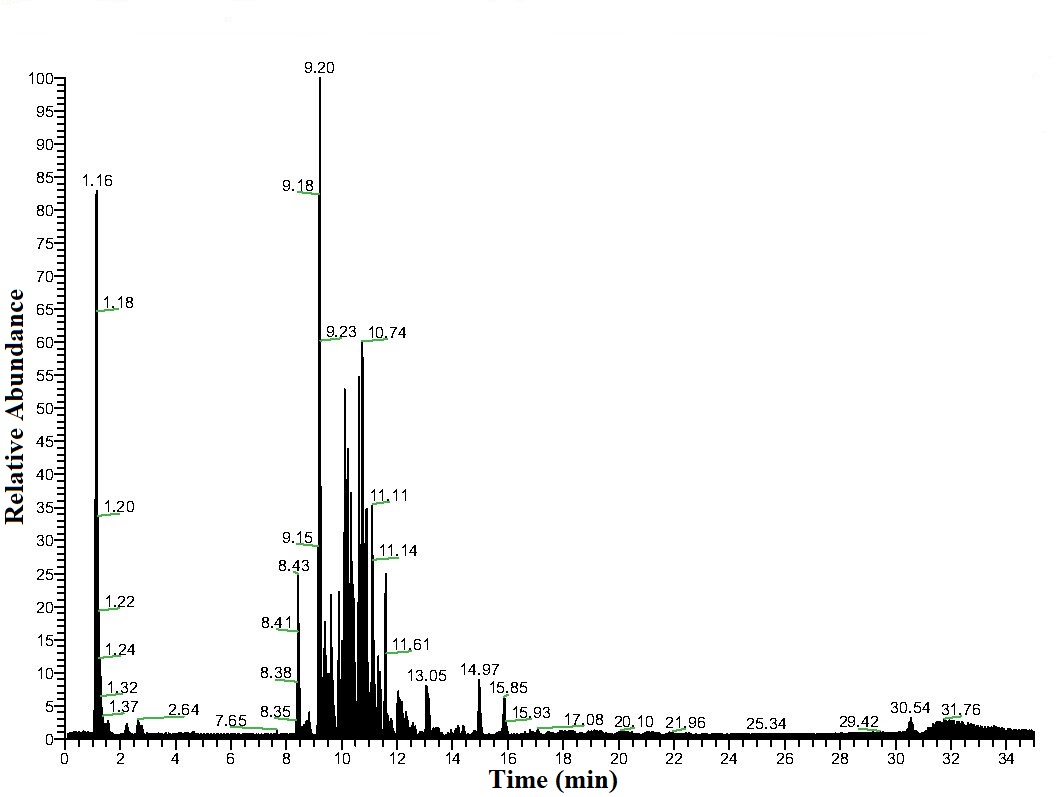

Supplement: Supplementary Figure 1 — Chromatogram in ESI positive (A) and negative ion modes (B) of QX. [file Image_1.jpg]

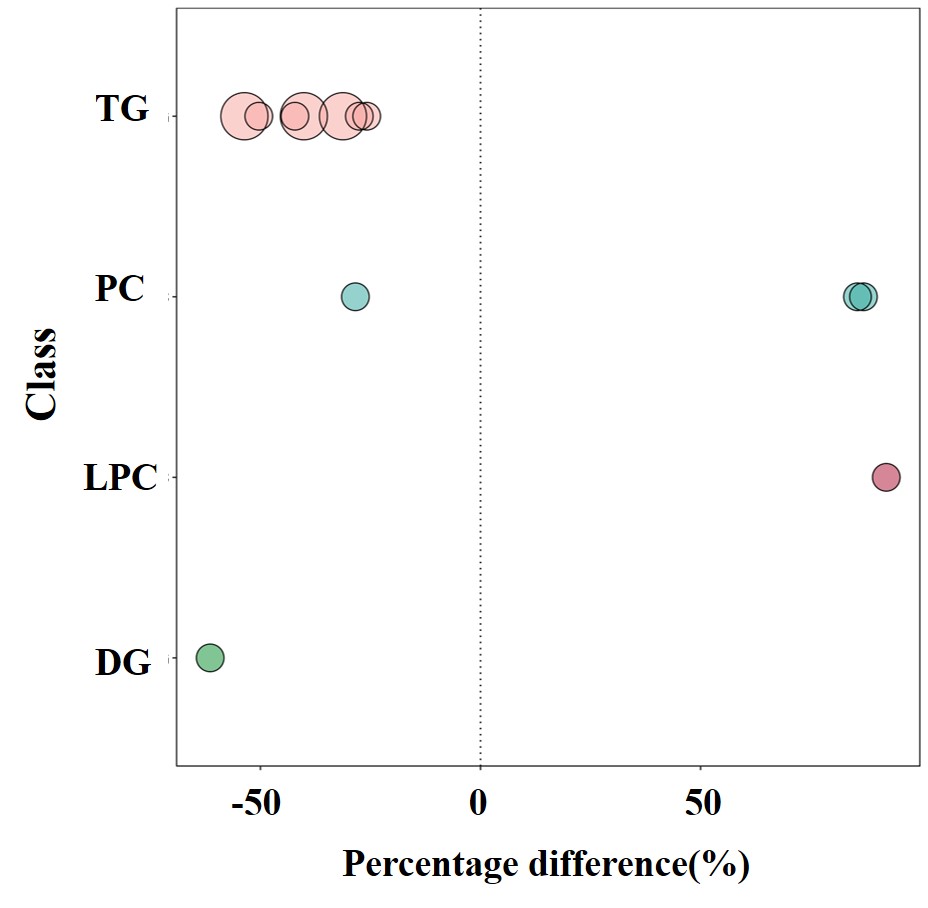

Supplement: Supplementary Figure 2 — (A) Secondary mass spectrometry of DG (18:2/18:2) (m/z 634.5405) generated fragmentation ion; (B) bubble chart, bubble in figure present significant difference of lipid molecules, different colors present different lipid subclasses. [file Image_2.jpg]

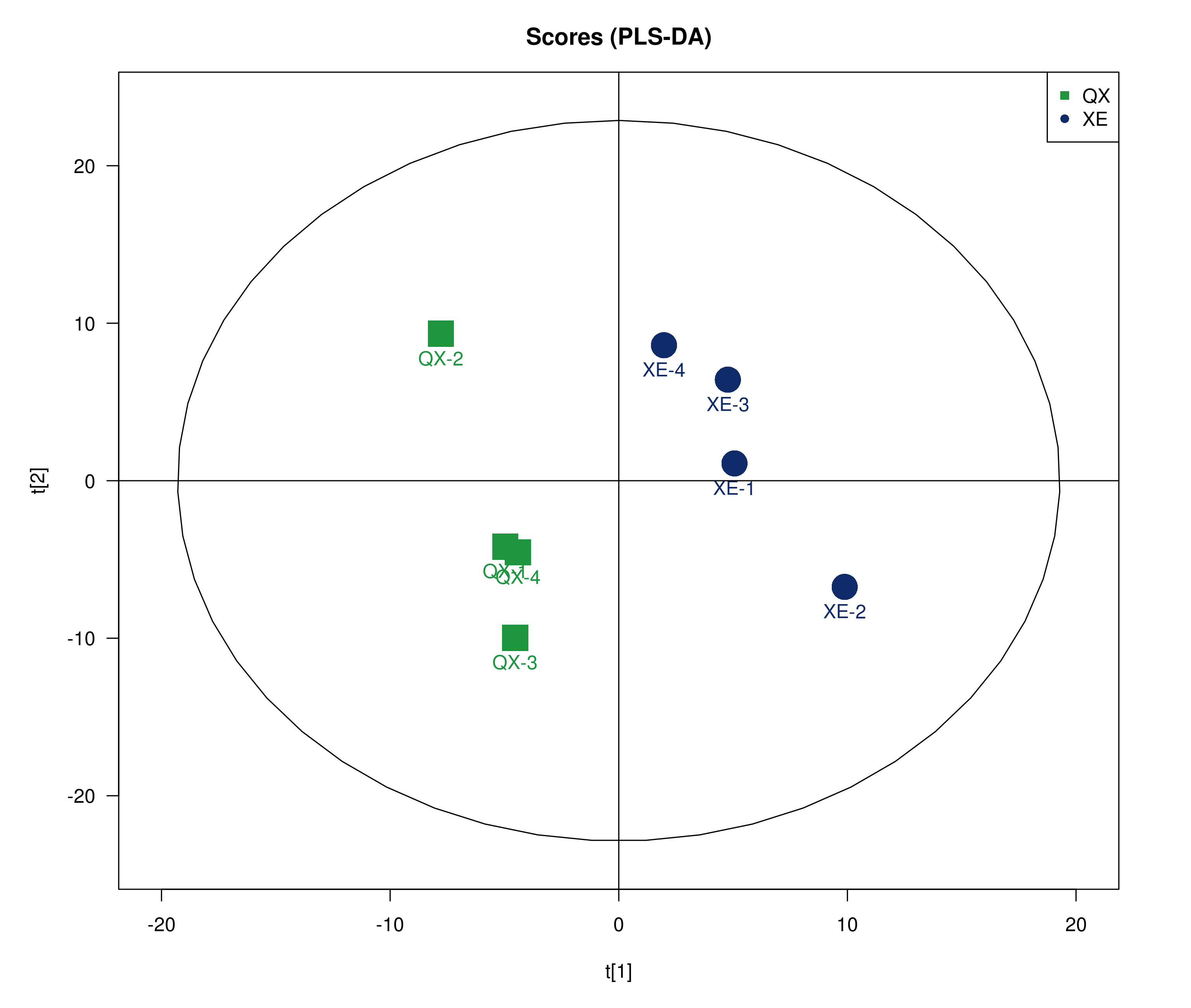

Supplement: Supplementary Figure 3 — (A) Score and loading plots generated from PLS-DA classifying the lipid of QX and XE. (B) The content of different numbers of carbon in TGs of two walnut varieties. [file Image_3.png]
